# Exosomes Derived from Adipose Stem Cells Enhance Bone Fracture Healing via the Activation of the Wnt3a/β-Catenin Signaling Pathway in Rats with Type 2 Diabetes Mellitus

**DOI:** 10.3390/ijms24054852

**Published:** 2023-03-02

**Authors:** Dong Zhang, Weidong Xiao, Changjiang Liu, Zheng Wang, Yuhang Liu, Yifeng Yu, Chao Jian, Aixi Yu

**Affiliations:** Department of Orthopedics Trauma and Microsurgery, Zhongnan Hospital of Wuhan University, Wuhan 430000, China

**Keywords:** exosome, ASCs, fracture, nonunion, osteogenesis, diabetes mellitus

## Abstract

Nonunion and delayed union are common complications of diabetes mellitus that pose a serious health threat to people. There are many approaches that have been used to improve bone fracture healing. Recently, exosomes have been regarded as promising medical biomaterials for improving fracture healing. However, whether exosomes derived from adipose stem cells can promote bone fracture healing in diabetes mellitus remains unclear. In this study, adipose stem cells (ASCs) and exosomes derived from adipose stem cells (ASCs-exos) are isolated and identified. Additionally, we evaluate the in vitro and in vivo effects of ASCs-exos on the osteogenic differentiation of bone marrow mesenchymal stem cells (BMSCs) and bone repair and the regeneration in a rat model of nonunion via Western blotting, immunofluorescence assay, ALP staining, alizarin red staining, radiographic examination and histological analysis. Compared with controls, ASCs-exos promoted BMSC osteogenic differentiation. Additionally, the results of Western blotting, radiographic examination and histological analysis show that ASCs-exos improve the ability for fracture repair in the rat model of nonunion bone fracture healing. Moreover, our results further proved that ASCs-exos play a role in activating the Wnt3a/β-catenin signaling pathway, which facilitates the osteogenic differentiation of BMSCs. All these results show that ASCs-exos enhance the osteogenic potential of BMSCs by activating the Wnt/β-catenin signaling pathway, and also facilitate the ability for bone repair and regeneration in vivo, which provides a novel direction for fracture nonunion in diabetes mellitus treatment.

## 1. Introduction

Over the last century, diabetes mellitus (DM) has developed into one of the most serious public health problems which leads to life threatening, disabling and costly complications, and seriously impairs life expectancy and quality worldwide [1,2]. In 2021, according to the data reported via the International Diabetes Federation, in excess of 1 in 10 adults currently have diabetes mellitus globally, and the number of patients with diabetes mellitus will continue to increase quickly in the future [3]. More seriously, diabetes mellitus often causes various difficult complications including stroke, coronary artery disease, neuropathy, kidney disease, hard-healing wounds and peripheral vascular disease [4,5]. Among these complications, the negative effects of diabetes mellitus on bone health have drawn much attention, and growing research has shown that diabetes mellitus may lead to osteoporosis, increase the risk of fracture and have an adverse impact on fracture healing [6]. When compared with non-diabetes, studies have shown that there is a higher risk of delayed union and nonunion, with diabetes patients experiencing double the time to heal a fracture [7]. Over the past few decades, despite significant investments in this field, there have been few satisfactory strategies introduced to enhance nonunion bone fracture healing [8,9]. Recently, mesenchymal stem cells (MSCs) derived from various sources, including bone marrow, umbilical cord and adipose tissue, have been seen as an effective tool for bone repair and regeneration [10,11]. Among the various sources of MSCs, ASCs have gradually been developed as a major approach in the field of repair and reconstruction based on their abundance and easier accessibility with minimally invasive procedures [12]. When compared with MSCs derived from other sources, ASCs hold great potential for proliferation and expansion [13]. In addition, they also have paracrine and immunomodulatory properties related to their specific secretome [14]. However, in spite of the above advantages, there are some associated risks bordering the underlying application of stem cell transplantation in the clinic to facilitate fracture healing, including immunosuppression, cell dedifferentiation and tumor formation [15,16]. Recently, a growing number of researchers have been holding the view that MSCs could promote tissue repair and reconstruction in a paracrine way via secreting bioactive factors. This viewpoint offers insight into the exploration of MSC derivates.

Exosomes are 50–150 nm in diameter and naturally released from almost all types of cells [17]. Recently, exosomes have opened a new door into bone repair and regeneration research, and have been broadly studied in the context of regeneration medicine [18,19]. Additionally, there is evidence that exosomes derived from serum, macrophages or MSCs are capable of being stably transferred into BMSCs, and are beneficial for the treatment of fracture healing [20,21,22]. More importantly, it has been confirmed that the application of transplanting exosomes displays similar therapeutic outcomes and functional properties as directly transplanted stem cells, but has less adverse effects such as immune rejection cell dedifferentiation and malignant transformation when using stem cells directly [23,24]. Among various cell types, ASCs represent an abundant MSC source, and are regarded as one of the most promising exosome sources due to a series of advantages [25,26,27]. Recent studies have demonstrated that ASCs-exos exhibit great potential in numerous exosome-based therapeutics for wound healing, cardiac injury and other reasons for tissue loss in DM [24,28,29,30]. 

Although these studies suggest that ASCs-exos are the key contributor for tissue repair, few research studies have focused on the therapeutic effects of ASCs-exos on promoting bone fracture healing in diabetes mellitus. Thus, this study aims to shed light on whether ASCs-exos can promote bone fracture healing in DM and affect the underlying mechanisms. Additionally, they may provide effective approaches for the treatment of impaired fracture healing in diabetes mellitus.

## 2. Results

### 2.1. Characterization of ASCs-exos

**Figure 1:**
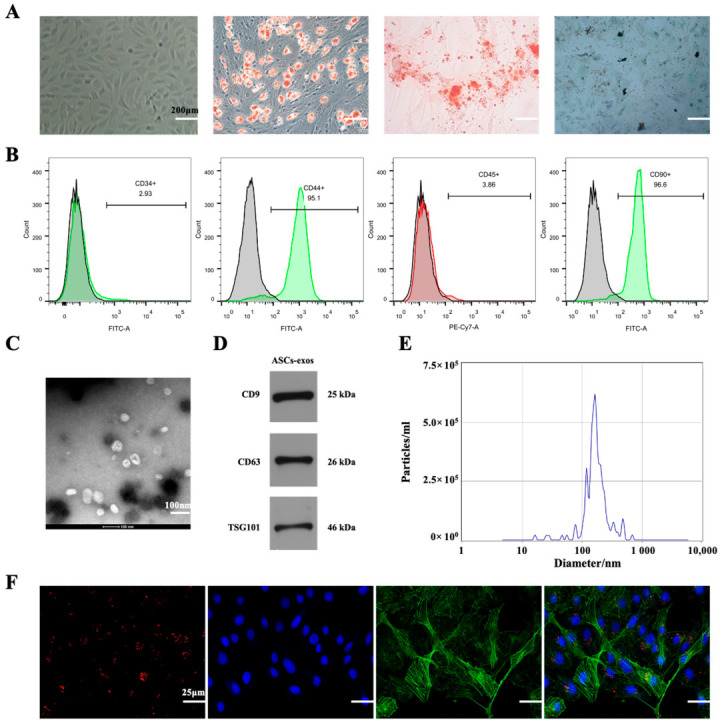
The identification of ASCs and ASCs-exos. (**A**) ASCs showed spindle-like morphology and multipotent differentiation potential, including adipogenic, osteogenic and chondrogenic differentiation. (**B**) ASCs were positive for CD44 and CD90, but negative for CD34 or CD45. (**C**) The observation of morphology using TEM. (**D**) The identification of particle size distributions using DLS. (**E**) Specific surface markers of exosomes including CD9, CD63 and TSG101 were identified using Western blotting. (**F**). The exosomes were marked with red fluorescence dye PKH26 and co-cultured with BMSCs; red fluorescence represents exosomes internalized by BMSCs.

ASCs were harvested from the subcutaneous fat in the groin of a rat, as described in the Materials and Methods section. The ASCs were identified by morphology, multipotent differentiation potential and flow cytometry. As shown in Figure 1A, ASCs displayed a shuttle-shaped morphology and could be induced to cause adipogenic, osteogenic or chondrogenic differentiation. Additionally, ASCs were positive for classical markers such as CD44^+^ and CD90^+^, but were negative for CD34^+^ and CD45^+^ (Figure 1B). All these results demonstrated that ASCs were successfully harvested. To identify the exosomes derived from ASCs, Western blotting, transmission electron microscopy (TEM) and dynamic light scattering (DLS) were performed. TEM analysis showed that the morphology of the ASCs-exos was cup-shaped (Figure 1C). The particle sizes of ASCs-exos were evaluated using DLS, and the results exhibited that their size distribution ranged from 30 to 200 nm (Figure 1D). Additionally, Western blotting results showed that ASCs-exos were positive for CD9, CD63 and TSG101, which are characteristic surface markers of exosomes (Figure 1E). All these data indicated that ASCs and ASCs-exos were successfully isolated. In addition, we further explored whether ASCs-exos can be internalized via BMSCs. In our following experiments, the results showed that the labeled ASCs-exos could be internalized by BMSCs (Figure 1F). 

### 2.2. ASCs-exos Promote BMSC Osteogenesis Differentiation In Vivo

**Figure 2:**
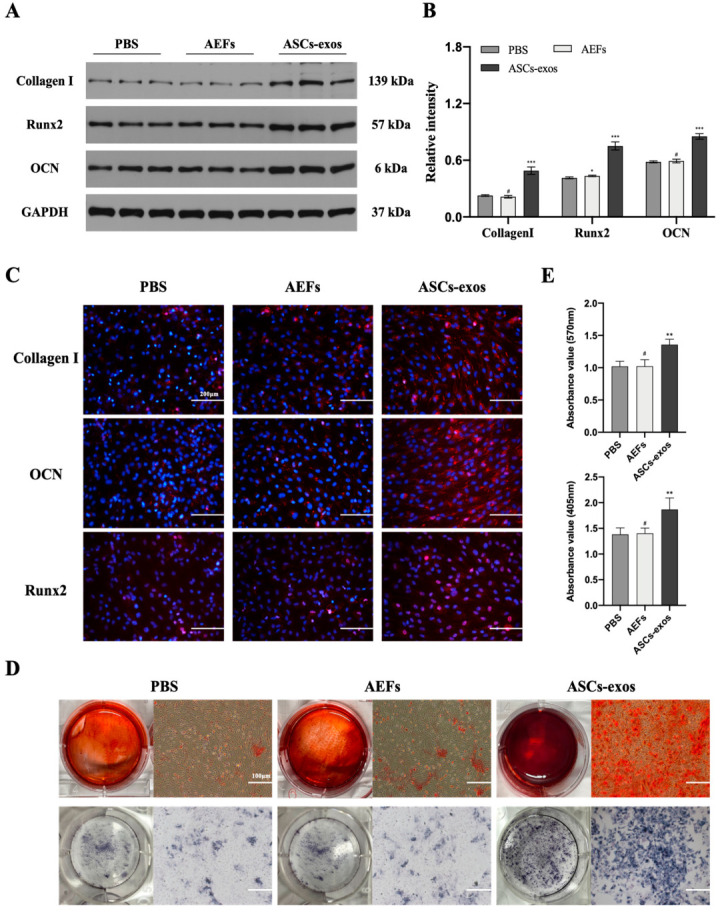
ASCs-exos facilitate BMSC osteogenesis differentiation in vitro. (**A**,**B**) The protein levels of Runx2, collagen I and OCN in BMSCs treated with PBS, AEFS or ASCs-exos were measured by Western blotting. (**C**) Immunofluorescence staining for collagen I, OCN and Runx2 in different groups. (**D**) The images of ARS and ALPS of BMSCs after 14 days of incubation with PBS, AEFS or ASCs-exos. (**E**) The statistical analysis of ARS and ALPS of BMSCs treated with PBS, AEFS or ASCs-exos for 14 days. Scale bars, 100 μm. Data are presented as the mean ± SD, and all experiments were repeated three times. * *p* < 0.05, ** *p* < 0.01, *** *p* < 0.001, # *p* > 0.05.

To further explore the effect of ASCs-exos on the ability for BMSC osteogenesis differentiation, BMSCs were co-cultured with PBS, ASCs-exos-free supernatant (AEFS) and ASCs-exos. As shown in Figure 2A,B, the protein expression of the osteogenesis-related genes was improved via ASCs-exos when compared with the control group. However, only the protein expression of Runx2 was improved via the AEFS in comparison with the PBS group. In addition, the immunofluorescence assay demonstrated that ASCs-exos promoted Runx2, collagen I and OCN protein expression in BMSCs on day 14 (Figure 2C). The results of alizarin red staining (ARS) and ALP staining (ALPS) showed that the proportion of mineralization was improved in the ASCs-exos group when compared with the PBS group, but there was no significant difference between the PBS group and the AEFS group (Figure 2D,E). In summary, these results demonstrated that ASCs-exos could promote osteogenic differentiation.

### 2.3. Successful Establishment of the Type 2 Diabetes Mellitus Rat Model 

**Figure 3:**
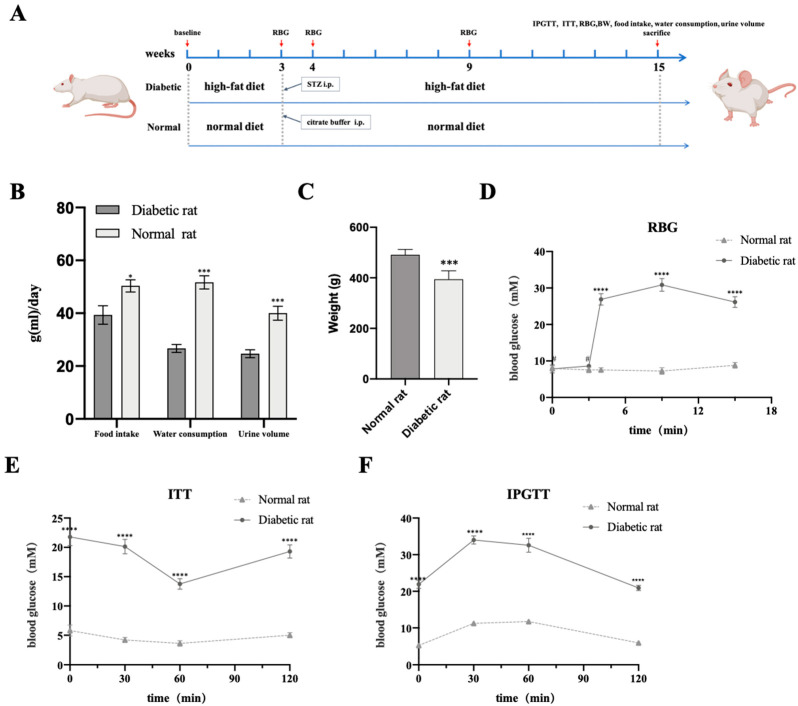
Assessment of T2DM rat models. (**A**) Procedure of animal operations. (**B**,**C**) Food intake, water consumption, urine volume and body weight (BW) of two groups at 12 weeks after STZ injection. (**D**) RBG at several special time points before fed HFD, before STZ injection, one week after STZ injection, 6 weeks after STZ injection and 12 weeks after STZ injection. (**E**,**F**) Blood glucose levels during ITT and IPGTT in two groups at 12 weeks after STZ injection. Data are presented as the mean ± SD (*n* = 18, per group). * *p* < 0.05, *** *p* < 0.001, **** *p* < 0.0001, # *p* > 0.05.

The results were similar to those from our previously published article, in which the same method was utilized to create the T2DM rat model. Our data demonstrated that all streptozocin (STZ)-induced rats on a high-fat diet (HFD) showed type 2 diabetes mellitus (T2DM). As shown in Figure 3B, food intake, water consumption and urine output in the T2DM group were significantly higher than that of the control group. On the contrary, rats in the T2DM group were thinner than those in the control group (Figure 3C). These characteristics are regarded as the typical symptoms of T2DM. Meanwhile, there was significance in two groups concerned with random blood glucose (RBG), and rats with T2DM had an RBG that was continually higher than 16.7 mmol/L (Figure 3D). Additionally, for the investigation of glucose tolerance and insulin sensitivity, we performed an insulin tolerance test (ITT) and intraperitoneal glucose tolerance test (IPGTT). As shown in Figure 3E, the level of blood glucose (BG) quickly declined in the normal group after insulin treatment, but was slow or did not reduce BG in the T2DM group within 30 min. In addition, the data in Figure 3F display how the rats with T2DM had hyperglycemia when compared with the control group for 120 min after glucose administration. In general, all these results suggest that we have successfully established a T2DM rat model with the characteristics of insulin resistance and hyperglycemia.

### 2.4. ASCs-exos Enhance T2DM-Delayed Fracture in Rat Model

**Figure 4:**
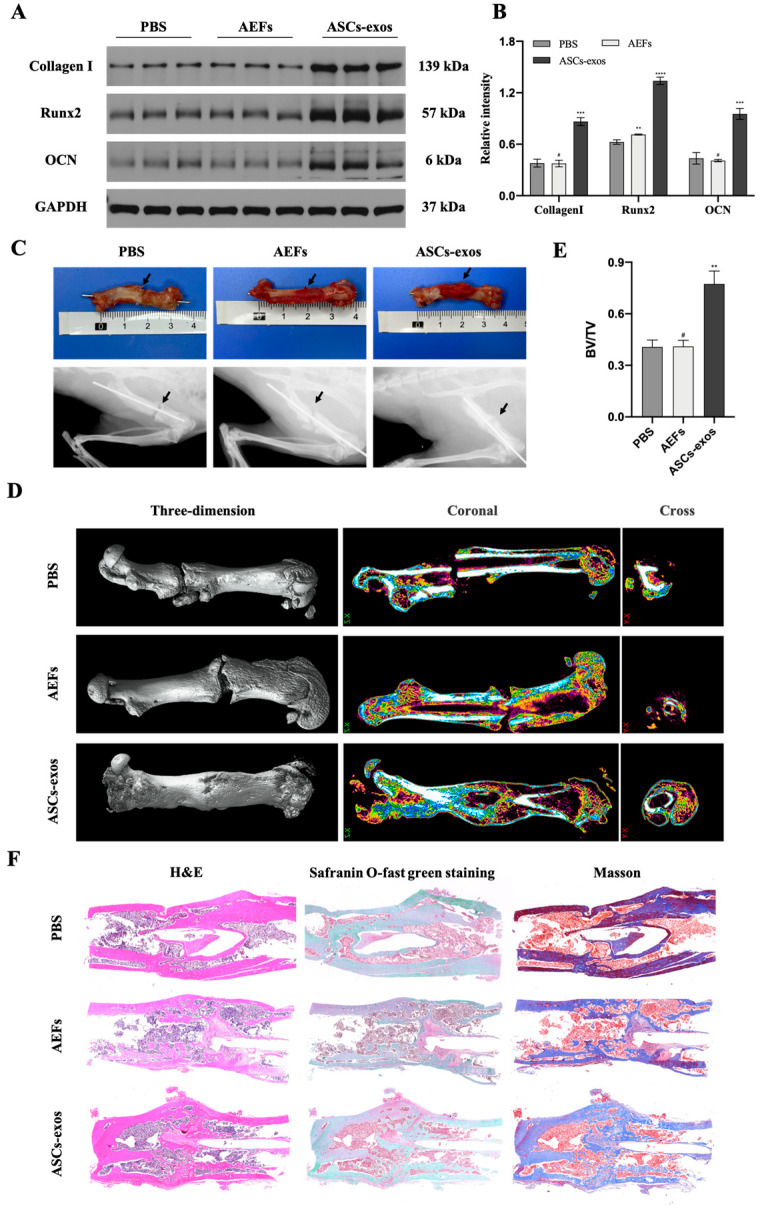
ASCs-exos accelerate fracture healing in T2DM rat models. (**A**,**B**) The protein levels of Runx2, collagen I and OCN at the fracture site treated with PBS, AEFS or ASCs-exos for 28 days were measured via Western blotting. (**C**) Representative digital images and X-ray images of fracture healing 28 days after surgery between PBS, AEFS and ASCs-exos groups. (**D**) Representative 3D, coronal and cross micro-CT images of fracture sites 28 days after surgery between PBS, S and ASCs-exos groups. (**E**) The statistical analysis of BV/TV according to micro-CT images 28 days after surgery among different groups. (**F**) Histology examination including H&E, safranin O-fast green and Masson staining of the femurs. Data are presented as the mean ± SD, and all experiments were repeated three times. ** *p* < 0.01, *** *p* < 0.001, **** *p* < 0.001, # *p* > 0.05.

The bone fractures in the DM rat model were treated with 600 μL ASCs-exos at a concentration of 200 μg/mL, and an equal volume of PBS and AEFS was applied around the fracture sites every three days after surgery to explore the effect of ASCs-exos on the treatment of fractures. The Western blotting results showed a significantly increased expression of Runx2, collagen I and OCN in the ASCs-exos group, and the AEFS group had no significant expression of osteogenesis-related genes compared with the PBS group (Figure 4A,B). Additionally, the digital imaging, X-ray imaging and micro-CT examinations were performed to evaluate the fracture repair at the fracture site. As shown in Figure 4C,D, the images demonstrated that, in comparison with the control group, the ASCs-exos group had a thicker callus volume and smaller fracture gap. Additionally, quantitative analysis of micro-CT data demonstrated that the bone volume/total volume (BV/TV) values of the ASCs-exos group were significantly increased in comparison with the control groups (Figure 4E). A histology examination 28 days after surgery suggested that there was a smaller hindrance in the fracture healing of the femur in ASCs-exos groups compared with other groups (Figure 4F). Collectively, these results implied that ASCs-exos improved the bone fracture healing in the T2DM rat model.

### 2.5. ASCs-exos Activated the Wnt3a/β-Catenin Signaling Pathway in BMSCs under High-Glucose Conditions

**Figure 5:**
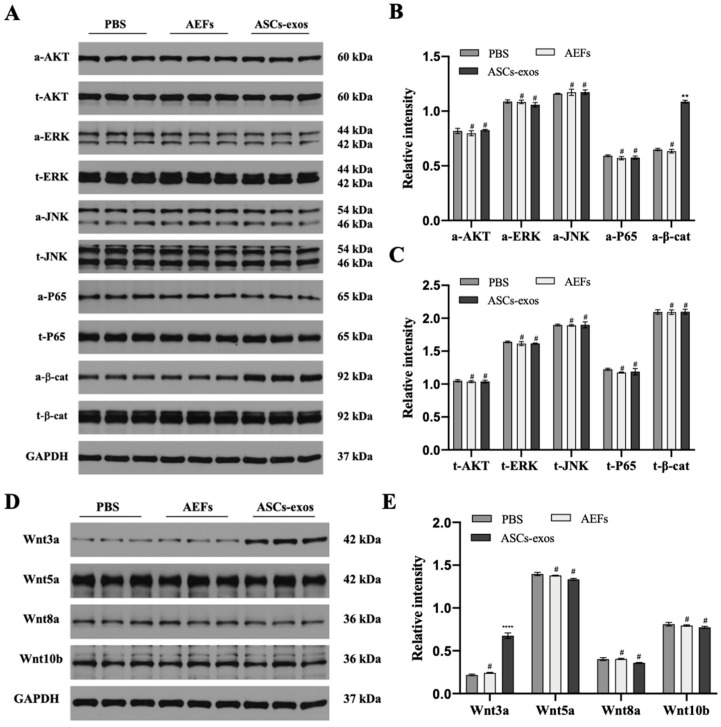
ASCs-exos activated the Wnt3a/β-catenin signaling pathway in vivo. (**A**,**B**) Comparison of signaling pathway-related protein levels in BMSCs treated with PBS, AEFS or ASCs-exos by Western blot analyses. (**C**,**D**) The protein expression of Wnt3a, Wnt5a, Wnt8a and Wnt10b was assessed by Western blot analyses. Data are presented as the mean ± SD, and all experiments were repeated three times. ** *p* < 0.01, **** *p* < 0.001, # *p* > 0.05.

To explore the specific signaling pathways via which ASCs-exos enhance the osteogenic differentiation of BMSCs, the common signaling pathways related to osteogenesis, including the PI3K/AKT signaling pathway, the MAPK signaling pathway, the NF-κB signaling pathway and the Wnt/β-catenin pathway, were observed via Western blotting. As shown in Figure 5A–C, there were no significant changes detected in the PI3K/AKT signaling pathway, the MAPK signaling pathway and the NF-κB signaling pathway. However, the protein expression of active β-catenin was significantly increased in BMSCs treated with ASCs-exos when compared with the control groups. The results revealed that the Wnt/β-catenin pathway might be involved in the enhancement of the BMSC osteogenic differentiation via ASCs-exos. Additionally, to further detect the role of canonical and noncanonical Wnt signaling, Western blotting was used to examine the expression of Wnt3a, Wnt5a, Wnt8a and Wnt10b. The expression of canonical Wnt3a was significantly upregulated in BMSCs treated with ASCs-exos, indicating that ASCs-exos modulate the Wnt signaling pathway in BMSC osteogenic differentiation (Figure 5D,E). 

### 2.6. The Activation of the Wnt3a/β-Catenin Signaling Pathway in BMSCs Treated with ASCs-exos under High-Glucose Conditions Can Be Inhibited via Dickkopf-Related Protein-1

**Figure 6:**
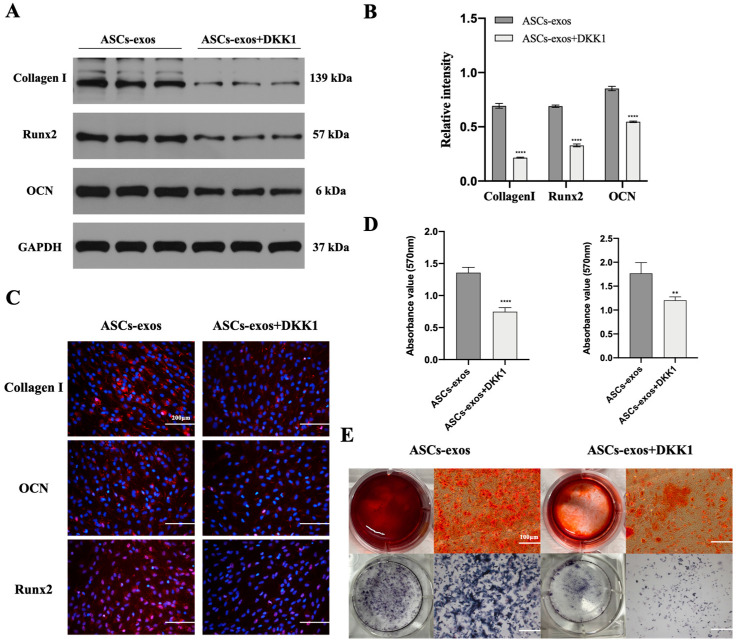
The positive effect of ASCs-exos on the osteogenic differentiation of BMSCs can be inhibited via DKK-1. (**A**,**B**) Decreased protein expression of collagen I, OCN and Runx2 due to the inhibition of Wnt3a/β-catenin signaling pathway via DKK-1. (**C**) Immunofluorescence staining for collagen I, OCN and Runx2. (**D**) Alizarin red staining and ALP staining in BMSCs after 14 days in different groups. (**E**) The statistical analysis of ARS and ALPS. Scale bars, 100 μm. Data are expressed as mean ± SD, and all experiments were repeated three times. ** *p* < 0.01, **** *p* < 0.001.

To further verify the involvement of the Wnt3a/β-catenin signaling pathway in BMSCs treated with ASCs-exos under high-glucose conditions, the activating effect of this signaling pathway on BMSC osteogenic differentiation induced via ASCs-exos was investigated. After treatment with Dickkopf-related protein-1 (DKK-1), an effective inhibitor of the Wnt/β-catenin signaling pathway, we found a nearly complete abrogation of the promotive effect on the protein levels of Runx2, collagen Ι and OCN induced by ASCs-exos (Figure 6A,B). Then, the results of the immunofluorescence assay further confirmed that the positive effect of ASCs-exos on the protein levels of Runx2, collagen Ι and OCN can be attenuated via DKK-1 (Figure 6C). Additionally, the mineralization levels were observed with ARS and ALPS, and the results displayed that they were also promoted via ASCs-exos while being inhibited by DKK-1; the effect of ASCs-exos could also be partially reversed via DKK-1 (Figure 6D,E). These results further suggest that there is a possibility of the Wnt3a/β-catenin pathway being involved in the BMSC osteogenic differentiation of the promoting effect of ASCs-exos. 

## 3. Discussion

As a common complication following DM, bone nonunion is an enormous challenge for patients and surgeons [31,32]. Additionally, researchers have investigated how the balance of bone homeostasis is disturbed during the process of bone nonunion in DM [33,34]. To address this disabling disease, various therapies including allografts, composite artificial bones, steam cell therapy and biological factors have been introduced [35]. Although various therapeutic approaches to fracture management have been explored via surgeons, the nonunion incidence has remained stable at around 10% of all patients with DM [36,37]. Thus, it is imperative to find efficient therapeutic strategies for the treatment of bone nonunion in DM.

Nowadays, exosomes have attracted much attention based on their important role in intercellular communications [23,38,39]. Additionally, mounting studies have demonstrated that exosomes from various types of cells have good performance on the tissue repair and regeneration via the delivering of various bioactive mediators, such as proteins, mRNAs or noncoding RNAs [22,40,41]. It is acknowledged that exosomes might have a significant effect on the pathological processes of DM, as well as related complications, from the perspective of cell-to-cell communication that occurs locally and between organs. For example, researchers have suggested that exosomes derived from adipose tissue play an important role in insulin resistance [42]; another study indicated that exosomes can be regarded as the key contributor to diabetic cardiac fibrosis and dysfunction, as they increase inflammatory and profibrogenic responses in fibroblasts [43]. As for bone repair and regeneration, Liao et al. reported that exosomes derived from BMSCs may prevent osteonecrosis of the femoral head via facilitating osteoblast proliferation, differentiation and osteogenesis, as well as angiogenesis [44]. Sun et al. suggested that osteoclast-derived exosomes are able to influence the process of osteoporosis by selectively inhibiting osteoblast activity [45]. Consistent with the above findings, another study suggested that exosomes derived from ASCs can facilitate bone regeneration by improving osteogenesis and angiogenesis [46]. Given the fact that ASCs are more accessible cell sources from which exosomes can be isolated and are able to produce higher amounts of exosomes than other cell types, such as endotheliocytes, fibroblasts or MSCs, we investigated whether ASCs-exos can improve bone fractures in DM or not. 

In our study, ASCs were successfully acquired and identified via morphology, and their multipotent differentiation potential was determined with flow cytometry; ASCs-exos were isolated from the supernatant of ASCs, and then they were identified via TEM, DLS and Western blotting. Similar to the evidence that exosomes are able to be internalized via target cells to modulate cell functions, our results have shown that exosomes derived from ASCs are taken up into BMSCs, and the osteogenesis ability of BMSCs was significantly improved via ASCs-exos in comparison with those in the PBS group. Similarly, we further displayed that the process of bone repair and regeneration in a DM rat model of fracture was enhanced via injecting ASCs-exos when compared with other groups. To investigate the molecular basis of osteogenic differentiation, we examined the effect of ASCs-exos on the common signaling pathways related to osteogenesis. Our results showed that the Wnt/β-catenin pathway might be involved in the enhancement of BMSC osteogenic differentiation via ASCs-exos. There is a growing amount of research that has demonstrated that the Wnt/β-catenin signaling pathway is regarded as a significant mediator of BMSC differentiation into osteoblasts via regulating β-catenin levels and subcellular localization [47,48]. Wnt/β-catenin signaling pathways include canonical and noncanonical approaches, in which canonical Wnt ligands promote osteogenesis, and noncanonical Wnt5a can inhibit canonical Wnt signaling [49]. In the canonical Wnt approach, the binding of canonical Wnt ligands, such as Wnt3a, Wnt8a and Wnt10b, to frizzled receptors on the cell surface results in the nuclear translocation of β-catenin, which ultimately binds with the TCF/LEF region to initiate the transcription of osteogenic genes such as Runx2 and OCN [50]. Furthermore, our findings further revealed that canonical Wnt3a was involved in the promotive effect of osteogenic differentiation in BMSCs induced via ASCs-exos. More importantly, our in vivo studies demonstrated that the promoting effect of ASCs-exos on the osteogenic differentiation of BMSCs can partially be blocked via DKK-1, which is regarded as an effective inhibitor of the Wnt/β-catenin signaling pathway. All these findings reveal that ASCs-exos have great potential in advancing the osteogenic differentiation of BMSCs via activating the Wnt3a/β-catenin signaling pathway, and provide a novel direction for the treatment of fracture nonunion in DM.

In addition, our data show that the AEFS held biological activity, including the upregulation of the protein expression of Runx2 in vitro. These results also suggest the potential usefulness of AEFS for particular situations.

However, there are some limitations to our current study. It is well known that exosomal therapeutic potential is mainly based on the content of different patterns of RNAs and proteins; therefore, further research is essential to explore the exact mechanism for improving bone fractures in DM. In addition, we may also need to further investigate the optimal dosage of ASCs-exos that has beneficial effects on bone fractures in DM. 

## 4. Materials and Methods

### 4.1. The T2DM Rat Model of Fracture Establishment and Treatment

All animal experiments were performed in compliance with the guidelines of the Institutional Animal Care and Use Committee (IACUC) of Wuhan University. Additionally, the experimental protocol was approved by the Committee on the Ethics of Animal Experiments of Wuhan University. All efforts were made to minimize animal suffering. After feeding for one week, the animals were accurately weighed and classified by cage (3 rats/cage), and 36 Sprague Dawley (SD) male rats were randomly distributed into 2 groups. Many studies have shown the procedures for creating a T2DM rat model [22,51]. After 3 weeks of an HFD containing 60% fat, 18 rats in the T2DM group were injected with STZ (40 mg/kg in citrate buffer). The control group with a normal diet received an equal volume of citrate buffer. To consecutively evaluate the blood glucose levels, blood samples were collected from the tail. According to the protocols, rats with more than three RBG samples >16.7 mmol/L were identified to have T2DM after 7 weeks. For the generation of a longstanding diabetes-related complication, animals were given free access to their original diets (the high-fat or control diet) for 12 weeks. For the assessment of the T2DM model, we measured the metabolic index including body weight, food intake, water consumption and volume of excreted urine at several time points, including before being fed the HFD and 12 weeks after STZ injection. In addition, RBG was observed at several special time points, which is shown in Figure 3A. At the end of observation, IPGTT was evaluated. Animals were fasted for 12 h and injected with 1.5 g/kg glucose. BG was measured at 0, 30, 60 and 120 min. Additionally, ITT was carried out by injecting the rats with 0.75 IU/kg insulin, and then BG was obtained at several special time points as was performed for IPGTT. Animals with an RBG below 10 mmol/L at any time were regarded as nondiabetic, and those with an RBG between 10 mmol/L and 16.7 mmol/L were excluded. At 12 weeks after STZ injection, rats in the T2DM group were used to establish the model of bone fracture. Additionally, rats in the control group were used for the isolation of ASCs.

Rats were positioned under general anesthesia with ketamine hydrochloride (60 mg/kg body weight) before surgery. To expose the femurs, a lateral incision was made along the proximal femur. The soft tissues including fascia and muscle were divided longitudinally; a transverse osteotomy of the mid-diaphysis of the femur was operated via an oscillating mini-saw. Then, a lateral parapatellar incision with the patella medially dislocated was used to expose the knee. After the femur intercondylar groove was adequately exposed via the full flexion of the knee joint, Kirschner’s wires were inserted to keep the fracture stably fixated at the center of the intercondylar groove, and the tip of the needle was run through the top of the greater trochanter of the femur. Finally, the incision was closed using a 5-0 nylon suture. All the rats were kept in a single cage. Unprotected weight bearing was allowed immediately. Based on the operation of X-ray examinations, the fracture sites of rats were located and marked on the skin. Then, 600 μL of ASCs-exos at a concentration of 200 μg/mL, as well as equal volumes of PBS and AEFS, was locally injected into the fracture site every three days after surgery. In addition, X-ray imaging, micro-CT, histological analysis and Western blot analysis of the fractured femurs were performed 28 days after operation. 

### 4.2. Cell Culture

ASCs were isolated and cultured following the methods previously described [52]. Eighteen SD rats with a normal diet were used in this part. The subcutaneous fat from the groin of the rat was harvested and washed two times with PBS. Adipose tissue was chopped using sterile operation scissors, which was followed by centrifugation at 1500 rpm for 10 min. Additionally, the supernatant was abolished, and the mixed collagenases were added into the precipitate. After digestion action for 40 min at 37 °C, the completed culture medium consisting of Dulbecco’s modified Eagle medium (DMEM, Gibco, CA, USA) high glucose, 10% fetal bovine serum (FBS, Serapro, CA, USA) and 1% penicillin/streptomycin was added to stop the reaction, and the mixture was filtered through a 70 μm filter. After another centrifugation at 1500 rpm for 8 min, the cell was resuspended in the completed culture medium and maintained with fresh culture medium supplemented on the 4th day. After 8 days of being cultured, ASCs were harvested for identification and analyzing the gain of exosomes. Briefly, an 80% confluence of ASCs was washed three times using PBS and cultured in the medium with exosome-depleted FBS. After 48–72 h, the supernatant was collected, centrifuged at 300× *g* for 10 min at 4 °C and then further centrifuged at 2000× *g* for 10 min at 4 °C to eliminate whole cells and cellular debris. Afterwards, the supernatant was recollected and centrifuged at 100,000× *g* for 6 h at 4 °C to pellet the exosomes. After gaining the precipitate that can be regarded as exosomes, the ASCs-exos-free supernatant was also collected for the following experiments. Additionally, the exosomes were washed using PBS to remove the contaminating proteins and continually ultracentrifuged at 100,000× *g* for 20 min at 4 °C. Finally, the exosomes were resuspended in PBS for the following experiments. The identification of exosomes was performed via TEM (HITACHI, HT7700) to confirm the morphology of the exosomes, DLS (Particle Metrix, Meerbusch, Germany), to analyze the diameter distributions and Western blotting to identify the specific exosome surface markers such as CD9, CD63 and TSG101. The rat BMSCs were from our laboratory, and the isolation and identification of rat BMSCs were described in our previous study [22,53]. A 200 g/L glucose solution was used to alter the glucose concentration of the medium. Before induced osteogenic differentiation, BMSCs were cultured in a medium that consisted of α-MEM supplemented with 10% FBS, 100 U/mL penicillin/streptomycin and 5 mM glucose. Additionally, BMSCs in the passages 3–5 were seeded into 6-well plates for osteogenic differentiation. To mimic the diabetic conditions in vitro, the glucose concentration of the osteogenic-induced medium used for the BMSCs was 30 mM. 

### 4.3. Identification of ASCs 

ASCs were identified via multipotent differentiation potential and flow cytometry. For adipogenic induction, ASCs were cultured in adipogenic induction medium (Cyagen Biosciences, Santa Clara, CA, USA). The adipocytes were stained with Oil red O staining on day 14. To induce osteogenic differentiation, a specific SD rat osteogenic induction medium (Cyagen Biosciences) was used. The cells were stained with ARS on day 14. For chondrogenic differentiation, ASCs were cultured for 2 weeks in chondrogenic induction medium (Cyagen Biosciences). On day 14, the cells were stained with toluidine blue to detect the secretion of sulfated glycosaminoglycans. Additionally, all of the media were replaced every 3 days. In addition, the identification of ASCs was measured via flow cytometry. ASCs were detected with antibodies against CD34 (Invitrogen, Waltham, MA, USA), CD45 (eBioscience, San Diego, CA, USA), CD44 (Invitrogen) and CD90 (BioLegend, San Diego, CA, USA). Results were analyzed via Flowjo software.

### 4.4. Exosome Uptake Assay

Based on the manufacturer’s protocol, PKH26 was used to label the exosomes for an exosome uptake assay. Briefly, the mixture of exosomes and PKH26 dye solution was incubated for 20 min at room temperature. Exosomes were obtained with centrifugation (110,000× *g*, 20 min, 4 °C). Additionally, BMSCs were seeded into a 35 mm confocal dish at the proper density and labeled with DAPI. Moreover, exosomes labeled with PKH26 were mixed with BMSCs labeled with DAPI. Finally, they were co-cultured for 12 h and observed using a fluorescence microscope.

### 4.5. BMSC Osteogenic Differentiation 

Passage 3 BMSCs were seeded into 6-well plates (2 × 10^5^ cells per well), which were precoated with 0.1% gelatin and incubated for 14 days using a specific osteogenic induction medium (Cyagen Biosciences). For evaluating the effect of ASCs-exos on osteogenic differentiation, 200 μL of ASCs-exos with a concentration of 200 μg/mL and equal volumes of PBS and AEFS was supplemented with the osteogenic induction medium and refreshed every three days. In addition, 500 ng/mL of DKK-1 was applied for investigating the involvement of the Wnt/β-catenin pathway in ASCs-exos by promoting the differentiation of BMSCs. To evaluate the level of osteogenic differentiation, the cells were stained with alkaline phosphatase (ALP) staining and alizarin red staining, and were collected for Western blotting on day 14.

### 4.6. Immunofluorescence Analysis

After BMSCs were induced for 2 weeks in different groups, the cells were fixed in 4% paraformaldehyde for 20 min at 25 °C, permeabilized for 30 min in 0.2% Triton X-100 and blocked for 30 min in 2% bovine serum albumin. Fixed cells were washed and incubated for 12 h with anti-Runx2 (1:300; CST), collagen I (1:300; Abcam) or OCN (1:300; PTG). After washing three times with PBS, cells were incubated with a fluorescence-conjugated secondary antibody (ASPEN) for 40 min, and the nuclei were stained with DAPI (Sigma) for 30 min. Finally, cells were observed via a fluorescence microscope.

### 4.7. ALP and Alizarin Red Staining

After BMSCs were induced for 14 days with different treatments, the cells washed two times via PBS and fixed with 4% paraformaldehyde for 30 min at room temperature were used for ALPS and ARS. A BCIP/NBT ALP kit (#C3206, Beyotime, Nantong, China) was used for ALPS. After the stained cells were washed using PBS three times, the BCIP/NBT substrate was utilized to treat BMSCs. The colorimetric changes of cells were imaged via microscopy. After removing the whole cells and cellular debris via centrifugation at 2000× *g* for 10 min at 4 °C, the supernatant was collected and distributed into each well of 96-well plates for an alkaline phosphatase activity assay. Subsequently, the absorbance of the samples was observed via a microplate reader at 405 nm. The quantification of ALPS was calculated by comparing the measured OD values against the standard curve. The 40 mM solution of ARS was used to stain the cells at room temperature for 30 min. Subsequently, the unbound alizarin red dye was eliminated, and stained BMSCs were washed three times using PBS and observed via microscopy. For ARS quantification, 10% acetic acid was added to the stained cells and they were incubated for 15 min at room temperature. After the collection of the supernatant via centrifugation at 2000× *g* for 10 min at 4 °C, 10% NH4OH was added and mixed into the supernatant. Finally, the absorbance was measured at 507 nm. Values were normalized to a calibration curve.

### 4.8. Radiographic and Histological Analysis 

The femurs of rats under different treatments were harvested and photographed on day 28 after surgery. To observe the fracture regions, the X-ray images were acquired using an X-ray imaging system for animals. After Kirschner’s wires were removed from the femurs, the fracture sites were scanned by using a micro-CT system (40 μm, 70 kV, 200 µA). Next, 3D structures and bone volume/total volume (BV/TV) of the fracture sites were obtained. After the samples were fixed with 4% paraformaldehyde and decalcified with 20% EDTA at 25 °C for 25 days, the tissues were then embedded in paraffin, and the samples were stained with Masson, H&E and safranin O-fast green staining. Finally, samples were observed using a microscope.

### 4.9. Western Blot Analysis

Western blotting was performed using previously described protocols. After the concentrations of protein were measured via BCA (Aspen), the protein was separated into equal amounts via SDS-PAGE (Beyotime Biotechnology, Shanghai, China), transferred into the PVDF membrane (Millipore, Burlington, MA, USA) and then incubated with 5% bovine serum albumin for 1 h at 25 °C. Next, the membranes were incubated overnight with primary antibodies specific for CD9 (Abcam), CD63 (Abcam), TSG101 (Abcam), Runx2 (Abcam), collagen I (Abcam), OCN (Santa), AKT (CST), ERK (CST), JNK (CST), p-65 (CST), β-catenin (CST), Wnt3a (Abcam), Wnt5a (Abcam), Wnt8a (Bioss), Wnt10b (Isbio) and GAPDH (Abcam). Then, samples were mixed with the secondary antibodies (1:2,000, 30 min). The membranes were incubated with Immobilon ECL reagent (Thermo Fisher Scientific, Waltham, MA, USA), and the bands were quantified via software.

### 4.10. Statistical Analysis

The data are represented as the mean ± SD, and were analyzed with GraphPad Prism 8.0. All the experiments were repeated at least three times. Student’s *t*-test was used to analyze the two independent groups. A value of *p* < 0.05 was considered to be statistically significant.

## 5. Conclusions

In conclusion, all these findings suggest that ASCs-exos are able to improve the osteogenic potential of BMSCs by activating the Wnt3a/β-catenin signaling pathway and facilitate the ability of bone repair and regeneration in vivo. Thus, our results indicate that ASCs-exos may be a promising therapeutic approach for enhancing bone fracture healing in DM.

## Data Availability

The datasets used and/or analyzed during the current study are available from the corresponding authors upon reasonable request.
